# Modeling the determinant of time to age at first marriage among women in Ethiopia using Cox models with mixed effects

**DOI:** 10.1186/s12978-022-01339-4

**Published:** 2022-01-31

**Authors:** Molalign Gualu Gobena, Yebelay Berelie

**Affiliations:** 1 Department of Statistics, Natural and Computational Sciences, Assosa University, P. Box 18, Assosa, Ethiopia; 2 Department of Statistics, Natural and Computational Sciences, Debre Markos University, P. Box 269, Debre Markos, Ethiopia

**Keywords:** Grouped data, Heterogeneity, Random effect, Shared frailty, Survival

## Abstract

**Background:**

Time to age at first marriage of women is the duration of time until the age at which they started living with their first partner. Time to age at first marriage is widely considered a proxy indicator for the age at which women begin to be exposed to the risks inherent in sexual activity. The purpose is to model the determinant of time to age at first marriage among women in Ethiopia using Cox models with mixed effects.

**Methods:**

The 2016 Ethiopian Demography and Health survey sample was selected using a two-stage cluster design. The data set in this study were obtained from the Demography and Health survey conducted in Ethiopia in 2016. In this study, we used Cox models with mixed effects.

**Results:**

Of all 15,683 women aged 15–49 years, 11,405 (72.72%) were married with the median and mean age at first marriage 17 years and 18 years, respectively. Cox frailty survival model showed that residence, educational level, occupation, work status of women& head education level of households were the most significant factors whereas religion, access to media and wealth index of a household of women were not significant factors at 5% level of significance. The significant clustering effect showed that heterogeneity among the regions on age at first marriage was present.

**Conclusions:**

The present study determined the duration of time until the age at first marriage and indicated relevant solutions for marriage-related problems of women aged 15–49 years in Ethiopia. Women residing in rural area of Ethiopia and had lower education level were married earlier. Therefore, programs to reduce the high rate of early marriage in Ethiopia should give attention to women education and women residing in rural area.

## Plain language summary

Time to age at first marriage of women is the duration of time until the age at which they started living with their first partner. African women are more likely to marry earlier than other continent women, which causes high fertility due to their long period of exposure to the risk of pregnancy. Even though Sub-Sahara Africa accounts for the highest rate of age at first marriage among countries in the Africa continent, comparably the case is very worse in Ethiopia. Furthermore, there is no study about the determinant of time to age at first marriage in Ethiopia using advanced models like Cox Model with Mixed effects. This model allows for the analysis of data with complex patterns of variability, with a focus on nested sources of variability. Very often it makes sense to use such a model to represent the variability within and between groups. For instance, in this study, our interest is not only knowing the significant effect of determinant factors on time to age at first marriage among women in Ethiopia but also the variability of time to age at first marriage within the region of Ethiopia and between regions of Ethiopia. We have used a dataset for this study from Ethiopia Demography and Health Survey which was conducted in 2016. The study helps to indicate relevant solutions for women's marriage-related problems (predominantly, women's reproductive health problems) in Ethiopia and it provides input for further studies in Ethiopia.

## Introduction

Time to age at first marriage of women is the duration of time until the age at which they started living with their first partner [1, 2]. Time to age at first marriage is widely considered a proxy indicator for the age at which women begin to be exposed to the risks inherent in sexual activity [2, 3]. A comparison of the median age at first intercourse with the median age at first marriage can be used as a measure of whether women engage in sex before marriage [2, 4]. The median age at first sexual intercourse for women aged 25–49 years is 16.6 years, which is very close to the median age at first marriage of 16.5 years. This suggests that Ethiopian women generally begin sexual intercourse at the time of their first marriage [2, 5]. In Ethiopia, marriage marks the point in a woman’s life when childbearing becomes socially acceptable. Age at first marriage has a major effect on childbearing because women who marry early have on average a longer period of exposure to the risk of pregnancy and give birth to a greater number of children over their lifetimes [2]. African women are more likely to marry earlier than other continent women, which causes high fertility due to their long period of exposure to the risk of pregnancy. Even though Sub-Sahara Africa accounts for the highest rate of age at first marriage among countries in the Africa continent, comparably the case is very worse in Ethiopia [6].

Time to age at first marriage of women have a vital role in determining the two public health indicators such as fertility and women period of exposure to the risk of pregnancy [7]. Therefore, modeling of the determinant for time to age at first marriage of women is the most desirable phenomena to briefly explain women reproductive health issue.

Previously, in Ethiopia, less concern is given for the broader women reproductive health implication of the determinant of time to age at first marriage of women [8]. From this aspect, conducting this study helps to identify whether the onset of reproduction occurs before the woman is adequately able to nurture her children and maintain her own health. In Ethiopia, there are poor and or lack of health facility and service. In the other side, very early age at first marriage and premarital first sex are common. Those, makes the case worst in Ethiopia [9]. Specifically, very early age at first marriage leads to marital instability and divorce, multiple partners; poverty, and subsequent drift into prostitution or paid domestic work [10]. The age at first marriage may also influence population growth, labor supply, consumption, wage rates, mortality, migration, and to some extent fertility [4]. Variation in the age of entry into marriage helps explain differences in fertility across populations and helps explain trends in fertility within individual populations over time. Women who marry early will have, on average; a longer period of exposure to the risk of pregnancy, often leading to higher completed fertility [5].

94% of all maternal deaths occur in low and lower middle-income countries like Ethiopia, Somalia, Sudan, etc. To clearly indicate, every day in 2015, approximately 353 women in Ethiopia died from preventable causes related to pregnancy and childbirth. Women ages 10–14 face a higher risk of complications and death as a result of pregnancy than other women [11]. In order to improve the reproductive health of women, it is important to identify the significance covariates/factors that affects the age at which women marry [7]. Hence, this study tried to address the women and their children public health problems like malnutrition, high rate of morbidity and mortality in Ethiopia by distinguish significant factors or covariates that are related to time-to-age at first marriage. It also estimate the variance of the random effect distribution for the data set in order to identify whether there is heterogeneity in time to age at first marriage of women among region of Ethiopia. Moreover, there is no study about the determinant of time to age at first marriage in Ethiopia using advanced models like Cox Model with Mixed effects. This model permits the analysts to account for the loss of independence that arises from the clustering of subjects in higher-level units [6].

In general, this study helps; to indicate relevant solutions for women marriage-related problems in Ethiopia, as inputs for modify women reproductive health policy and practice, and it provides input for further study in Ethiopia.

## Methodology

### Study design and setting

The study design of this study was a population based cross sectional study and data was obtained from 2016 Ethiopian Demographic and Health Survey (EDHS) collected from January 18 to June 27, 2016. The Survey was designed to provide estimates for the health and demographic variables of interest in nine geographical regions and two administration cities of Ethiopia. A total of 15,683 women of age 15–49 were interviewed in the survey.

### Variables in the study

The dependent variable is the time to age at first marriage. It is measured as the length of time from birth until the age at first marriage, which is measured in years. The independent variables considered in this study are the respondent's work status, religion, type of residence, head education level, women education level, head occupation, access to media, and wealth index.

### Method of data analysis

#### Survival analysis

Survival analysis consists of studies of the survival time of a subject (usually measured in days, weeks, months, or years), which is the time that elapses between the baseline and the moment an adverse event occurs, or the subject drops out of the trial. The survival times for subjects who dropped out of the trial are right-censored*. *The survival times of the subjects who remain in the trial until it ends are censored *as* well. In what follows, each uncensored observation is termed "death," regardless of whether death or a different adverse event has occurred. Denote by T the random variable representing the survival time of a subject. Let f(t)*,* t ≥ 0, denote the probability density function (pdf) of T*, *and let F(t*)* = P(T ≤ t)*, *t ≥ 0, be the cumulative distribution function (CDF) of T*. *The distribution of T is called the survival time distribution*.* The survival function*, *S(t)*, is* defined as the probability that a subject survives up to time *t* [12]*:*

2.1 $$S(t)= P (T > t) = {\int }_{t}^{\infty }f(x) dx =1-F(t), \mathrm{t}\ge \mathrm{O}$$

#### Median survival times

The median survival times to be the smallest value of t for which ≤ 0.5, that is, the time t where it jumps from a value greater than 0.5 to a value less than or equal to 0.5 [12].

#### Cox model with mixed effects

Multilevel or grouped data like individuals are nested within families, and families are nested within neighborhoods that are common across a wide range of fields of studies. This study also encountered such kinds of data. For instance, women aged 15–49 are nested within the region. As a result, it is two-level data. The inclusion of random effects into a Cox proportional hazard model shares many similarities with methods for the analysis of multilevel data with continuous, binary, or count outcomes. Cox proportional hazards model is enhanced through the incorporation of random effect terms to account for within-cluster homogeneity in outcomes. Applying the Cox proportional hazards regression to such grouped survival times leads to biased tests of statistical significance [13, 14]. Moreover, Cox’s model needs identically and independently distributed samples. Cox regression models with mixed effects do not assume as the observations are independent and allowed to apply for grouped data since it is one of the statistical models for multilevel survival analysis. The Cox regression model with mixed effects is said to be a frailty model when it is applied to two-level data.

Early frailty models incorporated subject-specific random effects to account for unmeasured subject characteristics that influenced the hazard of the occurrence of the outcome. These models were then extended to models that incorporate cluster-specific random effects to account for within-cluster homogeneity in outcomes. These models have been described as shared frailty models because the same random effect is shared by all subjects within the same cluster. As a result, the Cox regression model with mixed effects is said to be a shared frailty model when it is a model that incorporates cluster-specific random effects to account for within-cluster homogeneity in outcomes and particularly when it is applied to two-level data. For instance, in this study, women within the same region share the same random effect concerning marriage [15]. However, here we have used the special case of the Cox regression model with mixed effects, what we call the shared frailty model, to account for within-region homogeneity in the marriage of women. When random effects are incorporated in the Cox model, these random effects denote increased or decreased hazard for distinct classes. Suppose individuals are nested in one of G groups or clusters. A mixed-effects Cox regression model can be formulated as:

2.2 $${h}_{i}\left(t\right)={h}_{0}\left(t\right){exp}^{\left({x}_{i}\beta +{\alpha }_{j}\right)}$$

where ${\alpha }_{j}$ denotes the random effects associated with the j^th^ cluster. Rabe-Hesketh [16] used the term ‘shared frailty’ to denote the exponential of the random effect: exp(${\alpha }_{j})$. The random effect can be thought of as a random intercept that modifies the linear predictor, while the shared frailty term has a multiplicative effect on the baseline hazard function:

2.3 $${h}_{i}\left(t\right)={h}_{0}\left(t\right)\mathrm{exp}({\alpha }_{j}){exp}^{\left({x}_{i}\beta +{\alpha }_{j}\right)}$$

Cox regression models with mixed effects are characterized by the distribution of shared frailty terms. Different distributions have been proposed for the distribution of the shared frailty terms, including the gamma distribution, the log-normal distribution (the frailty terms will have a log-normal distribution while the random effects will have a normal distribution), positive stable frailty distributions and power variance function distributions. The first two appear to be the most commonly used. In the gamma frailty model, the cluster-specific random effects are distributed as the logarithms of independent, identically distributed gamma random variables, having variance$\theta$. In the log-normal frailty model, the cluster-specific random effects are distributed as the natural logarithms of independent, identically distributed normal random variables, having variance $\theta$. Generally, mixed-effects Cox regression models are used to model survival data when there are repeated measures on an individual, individuals nested within some other hierarchy, or some other reason to have both fixed and random effects [17, 18].

### Comparisons of models

Even though there are so many model selection criteria, AIC is the most familiar model selection criterium [19]. Therefore, here we used AIC criteria to compare two different Cox shared frailty models [i.e., Cox frailty survival model (log-normal frailty distribution) and Cox frailty survival model (gamma frailty distribution)]. Cox shared frailty model with the least AIC value is taken as the best-fitted model for the data set.

## Results

### Descriptive statistics

Of all 15,683 women aged 15–49, 11,405(72.72%) were married and the median & mean age at first marriage for women living in Ethiopia were 17 years and 18 years respectively, while the minimum and maximum age at first marriage observed were 10 years and 50 years respectively.

### Multivariable survival analysis for Cox models with mixed effects

In this study, we were doing multivariable survival analysis using Cox models with mixed effects. AIC criterion was used to compare models under Cox models with mixed effects. Accordingly, the Cox frailty survival model (log-normal frailty distribution) was selected as a better fit for the dataset compared to the Cox frailty survival model (gamma frailty distribution). Their AIC values are 193,779.4 and 193,779.7, respectively.

Analysis based on Cox frailty survival model (log-normal frailty distribution) showed that place of residence of women, one category of head occupation, education level of women, work status of women, and head education was significant at 5% level of significance (since their coefficient p-value < 5%). In contrast, the religion of women, access to media of women, and wealth index of a household were not significant at the 5% level of significance (since their coefficient p-value > 5%).

An odds ratio greater than 1 indicates that women with that category are more likely to extend their age at first marriage than women without that category. Therefore, women who attend primary school (ϕ = 1.3325), secondary school (ϕ = 2.2953), and higher education (ϕ = 2.8354) are more likely to extend their age at first marriage by a factor of 1.3325, 2.2953 & 2.8354 respectively than illiterate women (no education). Similarly, those women having a head that attends primary school, secondary school, and higher education are more likely to extend their age at first marriage by a factor of 4.7293, 4.7990, and 4.9841 respectively from those having an illiterate head (no education). In the same way, women who had work are 1.1319 times more likely to extend their age at first marriage than those who have not work (reference category).

Categories of significant covariates having an odds ratio less than 1 imply that women characterized by those categories of the same covariate are less likely to extend their age at first marriage than those women who are characterized by the reference category of the same covariates. For instance, women residing in a rural area of Ethiopia have married early than those residing in an urban area (ref) of Ethiopia (ϕ = 0.8880). Thus, those women who have laborer heads (ϕ = 0.9435) marry early than women who have a professional head. The variability (heterogeneity) among regions in Ethiopia with regard to the age at first marriage for women, which is estimated by the Cox frailty survival model (log-normal frailty distribution) was 0.9972. The test shows that there are a significant variation with regards to the age at first marriage of women among the region of Ethiopia (Table 1).

**Table 1:** Multivariable analysis using the Cox frailty survival model (log-normal frailty distribution)

Covariate	Coeff	St.err	P-value	ϕ
*Residence*				
Urban (ref)				
Rural	− 0.1188	0.0307	** < 0.001**	0.8880
*Education level*				
No Education (ref)				
Primary	0.2871	0.0524	** < 0.001**	1.3325
Secondary	0.8309	0.0495	< **0.001**	2.2953
Higher	1.0422	0.0520	< **0.001**	2.8354
*Religion*				
Catholic (ref)				
Orthodox	− 0.0516	0.1258	0.68	0.9498
Protestant	− 0.0834	0.1255	0.51	0.9200
Muslim	− 0.0148	0.1256	0.91	0.9854
Others	− 0.0929	0.1506	0.54	0.9113
*Wealth Index*				
Middle (ref)				
Poorer	− 0.0338	0.0353	0.34	0.9668
Poorest	− 0.0110	0.0323	0.54	0.9802
Richer	− 0.0363	0.0353	0.30	0.9644
Richest	− 0.0454	0.0337	0.18	0.9556
*Head education*				
No education (ref)				
Primary	1.5538	0.0326	< **0.001**	4.7293
Secondary	1.5684	0.0387	< **0.001**	4.7990
Higher	1.6063	0.0430	< **0.001**	4.9841
*Work status*				
No (ref)				
Yes	0.1239	0.0208	** < 0.001**	1.1319
*Access to media*				
Yes (ref)				
No	− 0.0273	0.0239	0.25	0.9730
*Head occupation*				
Professional (ref)				
Agriculturalist	− 0.0485	0.0255	0.057	0.9526
Laborers	− 0.0951	0.0400	**0.017**	0.9092
Business	− 0.0692	0.0373	0.064	0.9331
Others	− 0.0228	0.0431	0.60	0.9774
Random effect				
*Group name*	*Variance*			*P-value*
Region	0.9972			< **0.001**

## Discussion

This present study was conducted to determine the duration of time until the age at first marriage and indicated relevant solutions for marriage-related problems of women aged 15–49 years in Ethiopia. Analyses based on the Cox frailty survival model explain that women who are living in a rural area are more likely to marry early than in an urban area. This our result coincides with a study conducted in Ethiopia [20]. This is perhaps due to the fact that in Ethiopian context, girls residing in rural area are more likely to get married at an early age. Parents view marriage as a cultural arrangement to protect their daughter from sexual risks like adolescent pregnancy, rape, abduction, and also traditional faith that girls not successful in education as boys and hence marriage considered the best way to lead adult life for them. Furthermore, these women residing in rural area have less role in decision making for themselves and usually not consider their interest of marriage.

Work is an important factor for marriage. In this study we found that women who had work are more likely to marry later than those who have not and this result also supported by other studies [15] This is due to the fact that the economic advantage of women had a great role in delaying the age at first marriage and also they have more likely develop decision making power.

Education is a proximate predictor of women’s decision on their marriage time. We found that the increment of mean age at first marriage when the duration of schooling increases. Our result is other studies conducted in Nigeria [21]. It is well known that education affects the knowledge and attitudes of early marge and its consequences. Therefore, educated mothers can easily understand the problem of early marriage and also can control the family influence and decide herself.

In addition, in the current study, head of household educational level is also positively related to age at first marriage. Those women having better educated head of household had extended age at first marriage. The finding is also inline with other studies [22, 23]. The possible explanation might be the increment of the level of education for head gives to raise awareness for women and as a result of it leads to higher age at first marriage.

The finding of this study revealed that access to media, wealth index of the household, a religion of women, and some categories of head occupation were not significant factors at a 5% level of significance. However, our conclusion contradicts with other study findings conducted in Kenya, Nigeria [24] suggests that wealth index of households can improve age at first marriage,

### Strength and limitation

This is a population-based study that includes large sample data. Multiple confounding variables were evaluated using advance methodology. Our findings may not to be generalizable to all low- and middle-income countries, unless people have the same cultural norms for early marriage of girls and countries have similar socio-economic conditions. Since the data is observational data obtained from in the 2016 EDHS, there is a possibility of a recall bias when the participants answered the questions about the age at marriage.

## Conclusion

This study revealed that, the median age at first marriage for women aged 15–49 living in Ethiopia was 17 years and mean age at first marriage was 18 years. The key determinants that can have a direct influence on early marriage are education, residence and work status of women. The study also shows that women’s first age hetrogenity among region of Ethiopia. Therefore, the government and other concerned bodies should give prior attention for women residing in rural area of Ethiopia, women’s education access, and job opportunity for women/girls to delay early marriage and reduce its consequence in Ethiopia.

## Data Availability

The datasets and materials used in this study are available upon request to the corresponding author**.**

## Abbreviations

AIC: Akaike information criteria
CSA: Central Statistical Agency
EAs: Enumeration Areas
DHS: Demographic and Health Survey
EDHS: Ethiopian Demographic and Health Survey
